# Carboxylic Acid Production from Organic Waste: Integrating Substrate Composition, Reactor Configuration, Inoculum, and Future Perspectives

**DOI:** 10.3390/biotech15010016

**Published:** 2026-02-09

**Authors:** Ajay Thapa, Shiyu Fu, Joseph Sebastian, Onita Basu, Farah Hosseinian, Utsav Sharma, Dayanand Sharma, Abid Hussain

**Affiliations:** 1 Department of Civil and Environmental Engineering, Carleton University, 1125 Colonel by Drive, Ottawa, ON K1S 5B6, Canada; 2 Department of Chemistry, Carleton University, 1125 Colonel by Drive, Ottawa, ON K1S 5B6, Canada; 3 Department of Civil Engineering, Sharda University, Greater Noida 201310, India

**Keywords:** acidogenic fermentation, reactor configuration, substrate, inoculum, strategies, carboxylic acids

## Abstract

Acidogenic fermentation is a promising biotechnology for converting organic wastes into carboxylic acid (CA), which has significant commercial value and diverse applications in the food, chemical, pharmaceutical, and cosmetic industries. However, major challenges such as limited substrate hydrolysis and lower CA production hinder further development of this biotechnology towards full-scale implementation. This review provides a comprehensive overview of the current status of acidogenic fermentation, focusing on substrate composition, inoculum, and reactor design, along with potential strategies to overcome reactor-specific limitations and enhance CA production. It was found that the substrate composition, particularly its carbohydrate, protein, and lipid contents, strongly influences both CA production and yield. Specifically, carbohydrate-rich substrates yield higher CA production compared to protein- and lipid-rich substrates. These substrates have been investigated in different reactor configurations for CA production. Among them, the leachate bed reactor and anaerobic membrane bioreactor have demonstrated superior performance, achieving higher CA production with acetic and butyric acids as the dominant CA composition. These reactors are generally operated using three types of inocula: aerobic and anaerobic inoculum, enriched inoculum, and rumen microorganisms. Interestingly, rumen microorganisms are effective in degrading complex substrates, whereas enriched inoculum accelerates hydrolysis and acidogenesis processes within a shorter fermentation time. The findings presented herein will provide valuable information for addressing the challenges associated with acidogenic fermentation and lay the foundation for future research aimed at upscaling this biotechnology to a commercial scale.

## 1. Introduction

Growing environmental concerns and the transition toward a circular bioeconomy are driving increased attention to sustainable and eco-friendly chemical production. A promising approach is the valorization of organic wastes such as agro-industrial residues, forest residues, animal waste, and municipal solid waste for chemical production [1]. Acidogenic fermentation offers a viable pathway for converting organic wastes into value-added chemicals like carboxylic acids (CAs) through biological processes mediated by anaerobic microorganisms [2,3]. The CAs, such as acetic, propionic, butyric, valeric, and caproic acids, are fatty acids containing two to six carbon atoms. These CAs are vital chemicals with diverse applications in the food, chemical, pharmaceutical, and cosmetic industries, serve as precursors for bioplastic and biodiesel production, and act as carbon sources for nitrogen and phosphorus removal in biological wastewater treatment [1,4]. The average market price for these acids ranges from $0.89/kg for acetic acid, $2.20/kg for propionic acid, $2.55/kg for butyric acid, $2.75/kg for isobuytric acid, $4.63/kg for valeric acid, and $5.20/kg for caproic acid [1,4,5]. Currently, their production mostly depends on non-renewable petrochemicals through energy-intensive chemical synthesis processes, which contribute significantly to greenhouse gas emissions [2]. Consequently, the bioproduction of CAs from organic waste has attracted increasing attention due to concerns over climate change, fossil fuel depletion, and sustainable waste management. In this context, this approach is particularly relevant for addressing pressing environmental concerns by promoting sustainable waste management, especially in light of the projected 42% increase in organic waste generation by 2050 [1,6].

However, the major limitations of acidogenic fermentation are low substrate hydrolysis and reactor-specific constraints, which consequently hamper CA production [1,4]. Additionally, it generates a mixture of CAs with varying compositions, necessitating further downstream recovery and purification processes. This variability is largely associated with differences in the composition of organic wastes, particularly carbohydrates, proteins, and lipids [7,8]. To address these limitations, extensive research has focused on enhancing CA production through pretreatment methods, optimization of operational parameters, and the development of downstream recovery and purification methods [8,9].

Previous reviews have primarily summarized these strategies, along with the associated microbial communities and metabolic pathways, underscoring the importance of an integrated approach for enhancing CA production and realizing its commercial potential [2,8,9,10]. However, a comprehensive review that systematically integrates substrate composition, inoculum selection, and recent advances in reactor configurations, along with strategies to overcome reactor-specific limitations, remains lacking. To address this gap, this review provides a detailed summary of the following areas: (1) substrate composition and metabolic pathways involved in CA production, (2) state-of-the-art reactor configurations and strategies to overcome reactor limitations and enhance CA production, (3) inoculum selection, and (4) challenges and future perspectives for improving CA production.

## 2. Substrate Composition, Metabolic Pathways, and Their Impact on CA Production

Acidogenic fermentation is an emerging biotechnology for converting organic wastes into CAs [1]. Various organic wastes such as food waste, waste activated sludge (WAS), wastewater, potato peel, and animal manure have been extensively investigated as potential substrates for CA production [8,9]. Based on their biochemical characteristics, substrates can be broadly classified into carbohydrates, proteins, and lipids. Each of these components exhibits distinct metabolic pathways, hydrolysis processes, and CA production profiles, which are discussed in the following sections.

### 2.1. Carbohydrate-Rich Substrate

Carbohydrate-rich substrates are commonly utilized for CA production due to their higher biodegradability. They can be broadly categorized into readily degradable carbohydrates and complex carbohydrates.

#### 2.1.1. Readily Degradable Carbohydrate

Readily degradable carbohydrates that have been explored for CA production include cheese whey, maize silage, pulp and papermill effluent, and potato peels. During acidogenic fermentation, hydrolysis is often considered the rate-limiting step because of the substrate’s rigid and complex structure [1]. The hydrolysis products are a crucial component required for the CA production. When readily degradable substrates such as glucose were used, hydrolysis proceeded rapidly, resulting in a CA production efficiency of 97%, indicating an almost complete conversion of soluble chemical oxygen demand (SCOD) to CA [10]. Similarly, nearly 100% conversion of SCOD into CA was obtained when using cheese whey permeate and paper mill effluent, resulting in maximum CA yields of 0.60 gCOD_CA_/gSCOD and 0.59 gCOD_CA_/gSCOD, respectively [11]. Interestingly, the CA yield varies significantly even among readily degradable substrates. For example, the fermentation of cheese whey produced a CA yield of 0.71 gCOD_CA_/gSCOD, which was around 10% lower than that of maize silage (0.78 gCOD_CA_/gSCOD) [12]. The higher CA yield obtained from maize silage was attributed to its high starch content, which facilitated efficient hydrolysis and acidogenesis. These findings suggest that substrate composition plays an important role in determining the efficiency of CA production. This result was further supported by Zhang et al. [13], who compared the CA production potential of four different vegetables: potato peels, carrots, celery, and Chinese cabbage. Among these, potato peels showed the highest CA yield, which was 40.1%, 21.5%, and 124.9% higher than those obtained from carrots, celery, and Chinese cabbage, respectively. Furthermore, the CA composition was diverse in potato peels, consisting of acetic, propionic, butyric, valeric, and hexanoic acids. In contrast, butyric acid dominated the CA composition in carrots, while equal proportions of acetic and propionic acids were observed in celery and Chinese cabbage. Overall, these studies highlight that even when substrates are easily hydrolyzed, their composition can significantly influence both CA production and composition.

#### 2.1.2. Complex Carbohydrates

Complex carbohydrates, such as lignocellulosic biomass derived from agro-industrial residues, including brewer’s spent grain (BSG), corn stover, and rice straw, have been studied as potential substrates for CA production (Table 1) [14,15]. Lignocellulosic biomass consists of cellulose, hemicellulose, and lignin, which are tightly interconnected by covalent and hydrogen bonds. Although it is rich in carbohydrates, the presence of lignin makes it highly resistant to degradation, thereby hindering the hydrolysis of cellulose and hemicellulose for CA production. To overcome this barrier, various pretreatment methods are often employed to disintegrate lignin and improve accessibility of cellulose to microorganisms [1,14]. For instance, Castilla-Archilla et al. [16] applied a thermal diluted acid pretreatment on BSG, which facilitated the release of simple sugars such as glucose, xylose, and arabinose. This approach resulted in the highest CA production of 16.9 g COD/L, corresponding to an acidification degree of 93%, with acetic and butyric acids as the dominant CA composition. In contrast, a study using raw BSG (without pretreatment) reported a higher CA production of 35.5 g COD/L with an acidification level of 81% [17]. The improved CA production was attributed to the physical milling of the BSG prior to fermentation, which increased cellulose accessibility to microorganisms. Likewise, corn stover, a major agro-industrial residue, has been extensively studied for CA production. It primarily consists of about 70% cellulose and hemicellulose, and 15 to 20% lignin, making it a promising substrate due to the fermentable nature of its polysaccharide components [18]. For example, wet explosion pretreatment applied to corn stover resulted in CA productions of 22.8 g COD/L at 2.5% total solids (TS) and 40.8 g COD/L at 5% TS [18]. Similarly, Song et al. [19] reported a CA production of 10.5 g COD/L from hydrothermally pretreated corn stover. Overall, these findings underscore the huge potential of complex carbohydrate-rich substrates for CA production and emphasize the vital role of pretreatment in enhancing substrate biodegradability.

### 2.2. Protein-Rich Substrate

Protein-rich substrates such as tofu, egg white, tuna waste, dairy wastewater, WAS, and slaughterhouse wastewater have been employed for CA production. The anaerobic degradation of proteins is relatively slower than that of carbohydrates due to their complex molecular structure [8,23,25,30]. Furthermore, microorganisms cannot directly metabolize proteins and therefore rely on extracellular proteases to hydrolyze them into amino acids and peptides. After that, these simpler organic compounds are fermented into CAs and ammonium [25,30]. Consequently, hydrolysis is often considered the rate-limiting step when using substrates with higher protein contents [8,25]. However, Duong et al. [30] reported that protein hydrolysis occurred five times faster than acidification of the hydrolysis products, suggesting that acidification, rather than hydrolysis, could be the rate-limiting step during protein degradation. The degradation of proteins can be accelerated either by applying enzymatic pretreatment or by operating under alkaline pH conditions [24,31,32]. For instance, Wan et al. [31] applied an enzymatic cocktail (consisting of protease, pectinase, and laccase) pretreatment to WAS and observed maximum SCOD and CA production of 12 g/L and 10.6 g COD/L, respectively, which was significantly higher (*p* < 0.05) than those obtained from unpretreated WAS during 16 days of fermentation. Similar improvements in CA production have been reported when enzymatic pretreatment was applied to WAS [33,34,35]. The CA production and composition from various protein-rich substrates are presented in Table 1. Among these substrates, tuna waste produced the highest CA production of 30.6 g COD/L at an optimal pH of 8, with acetic, propionic, butyric, and valeric acids as the dominant components. Similarly, egg white and tofu have been studied for CA production [25]. The optimal CA yields were reported as 0.46 g/g VS for tofu with hydrothermal pretreatment and 0.26 g/g VS for egg white without hydrothermal pretreatment. Tofu was capable of directly producing CAs via the Stickland reaction, while egg white was simultaneously converted into lactate and CAs. The CA composition varied significantly between the two substrates: for tofu, acetic acid was dominant (56% of the total CA), followed by propionic (16%), butyric (10%), and valeric acids (18%). In contrast, egg white produced a uniform CA distribution, with each of these CAs consisting of 25% of the total CA. Overall, protein-rich substrates represent a promising feedstock for CA production due to their high organic content. However, their complex structure and the accumulation of ammonium can inhibit microbial activity and ultimately lead to process failure. Therefore, applying potential strategies such as enzymatic and hydrothermal pretreatments, along with maintaining alkaline pH conditions, can enhance hydrolysis and improve CA production from protein-rich substrates.

### 2.3. Lipid-Rich Substrate

Lipid substrates such as crude glycerol, olive mill solid waste, and olive oil mill effluent have been utilized for CA production. Compared to carbohydrate- and protein-based substrates, lipid-rich substrates are generally less preferred for CA production due to their slower hydrolysis rates and the inhibitory effects of their intermediate products [8,27]. The hydrolysis of lipids produces glycerol and long-chain fatty acids (LCFAs). The accumulation of LCFAs on bacterial cell surfaces hampers nutrient transport and enzymatic activity, ultimately leading to process failure [36,37]. To mitigate these challenges, the use of enriched microbial consortia that are functionally active and acclimatized to LCFA toxicity has been proposed as a promising approach to enhance hydrolysis and CA production from lipid-rich substrates [8,37]. For instance, Saha et al. [36] applied enriched microbial consortia for the treatment of fat, oil, and grease (FOG), achieving 90% LCFA removal along with the production of 1.61 mM butyric acid and 0.90 mM caproic acid. Despite the high organic content, lipid-rich substrates have been underexplored for CA production, with only a few studies reported to date (Table 1).

### 2.4. Metabolic Pathways in Acidogenic Fermentation for CA Production

**Figure 1:**
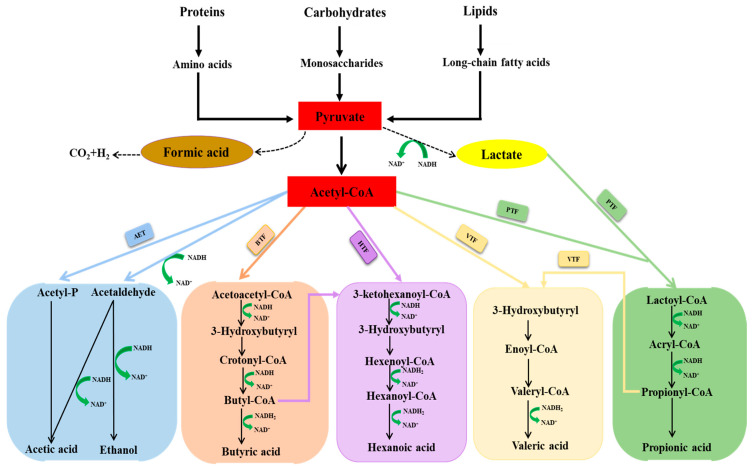
Schematic diagram of metabolic pathways involved in acidogenic fermentation (AET:—acetate-ethanol type fermentation; PTF: —propionate-type fermentation; BTF:—butyrate-type fermentation; VTF:—valerate-type fermentation; HTF:—hexanoate-type fermentation).

In acidogenic fermentation, complex substrates such as carbohydrates, proteins, and lipids first undergo hydrolysis, during which they are broken down into simpler organic compounds—monosaccharides, amino acids, and long-chain fatty acids, respectively. Hydrolytic bacteria belonging to the phyla of Firmicutes, Proteobacteria, Bacteroidetes, Actinobacteria, and Chloroflexi secrete various extracellular enzymes, including hydrolases, amylases, lipases, and proteases, which facilitate the release of these organic compounds [1,38]. Following hydrolysis, the resulting organic compounds are utilized by acidogenic bacteria and converted into pyruvate, which subsequently enters various metabolic pathways producing CAs, ethanol, hydrogen (H_2_), and carbon dioxide (CO_2_) [1]. The acidogenic bacteria belonging to the phyla of Bacteroidetes, Firmicutes, Proteobacteria, Actinobacteria, and Chloroflexi are involved in both hydrolysis and acidogenesis [1,39]. The major metabolic pathways involved in acidogenic fermentation are acetate-ethanol type, propionate-type, and butyrate-type, respectively, as illustrated in Figure 1 [1,40]. In the acetate-ethanol pathway, acetic acid is primarily produced from acetyl-CoA via acetyl-P and acetaldehyde (Figure 1). Additionally, acetic acid can also be generated through the syntrophic oxidation of propionic acid, butyric acid, or ethanol [1,40]. The predominant functional bacteria responsible for acetic acid production include *Bacteroides*, *Bifidobacterium*, *Tissierella*, *Acholeplasma*, and *Clostridiales* [1,2,38,41]. Propionic acid is produced through the reduction of pyruvate via acetyl-CoA and lactate intermediates. Specifically, pyruvate is first reduced to lactate by lactate dehydrogenase, after which lactate is converted to propionic acid by propionate dehydrogenase (Figure 1) [40]. The key propionic acid-producing bacteria include *Propionibacterium*, *Parabacterpide*, *Paludibacter*, *Prevotella*, and *Clostridium* [1,2,38,41]. Similarly, butyric acid is synthesized from the reduction of pyruvate via acetyl-CoA through a series of intermediates, including acetoacetyl-CoA, 3-hydroxybutyryl, crotonyl-CoA, and butyl-CoA (Figure 1). The dominant functional acidogenic bacteria associated with butyric acid include *Pseudomonas*, *Corynebacterium*, *Enterococcus*, *Clostridium Sensu Stricto 1*, and *Bacteroides* [2,42,43]. All these metabolic conversions are driven by specific enzymes produced by anaerobic bacteria during acidogenic fermentation. The key enzymes responsible for acetic acid production are acetate kinase and phosphotransacetylase, whereas oxaloacetate transcarboxylase and CoA transferase are mainly involved in the propionic acid production [38]. Similarly, phosphotransbutyrlase and butyrate kinase plays vital role in butyric acid production [40]. Therefore, elucidating the microbial and enzymatic mechanisms is essential for developing robust systems and channeling fermentation processes toward targeted CA production.

## 3. Reactor Configurations Applied for Acidogenic Fermentation

**Figure 2:**
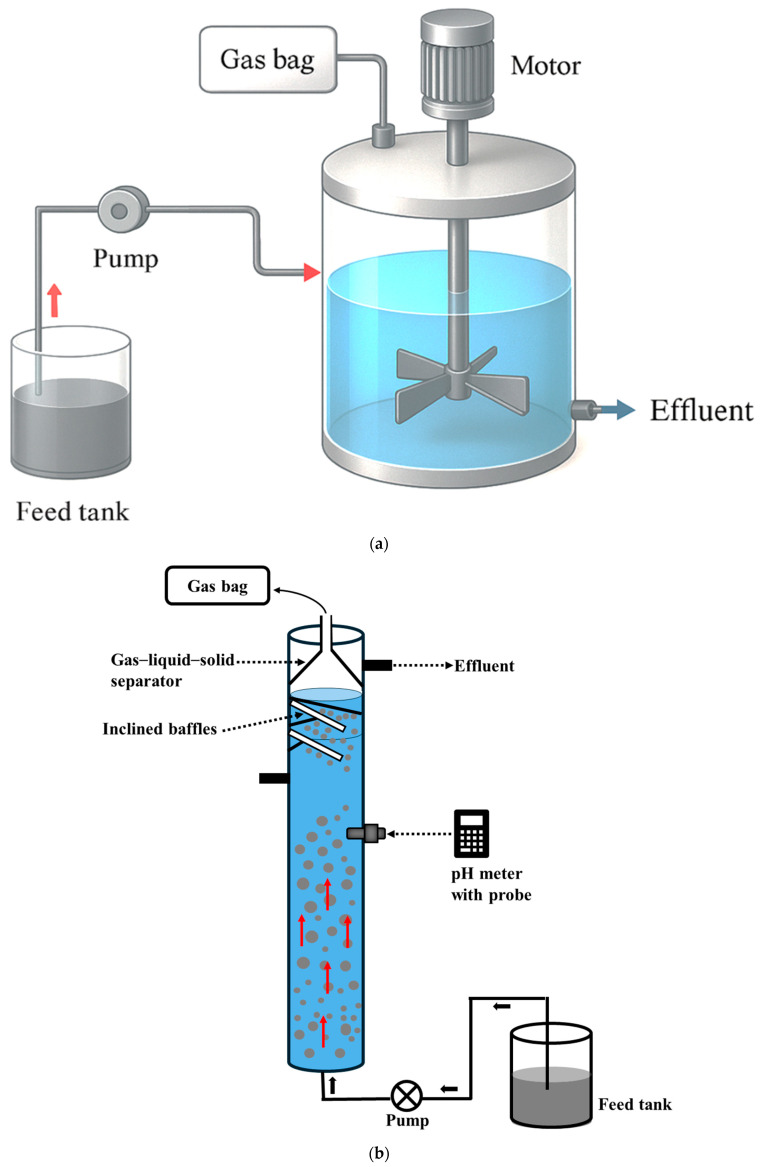

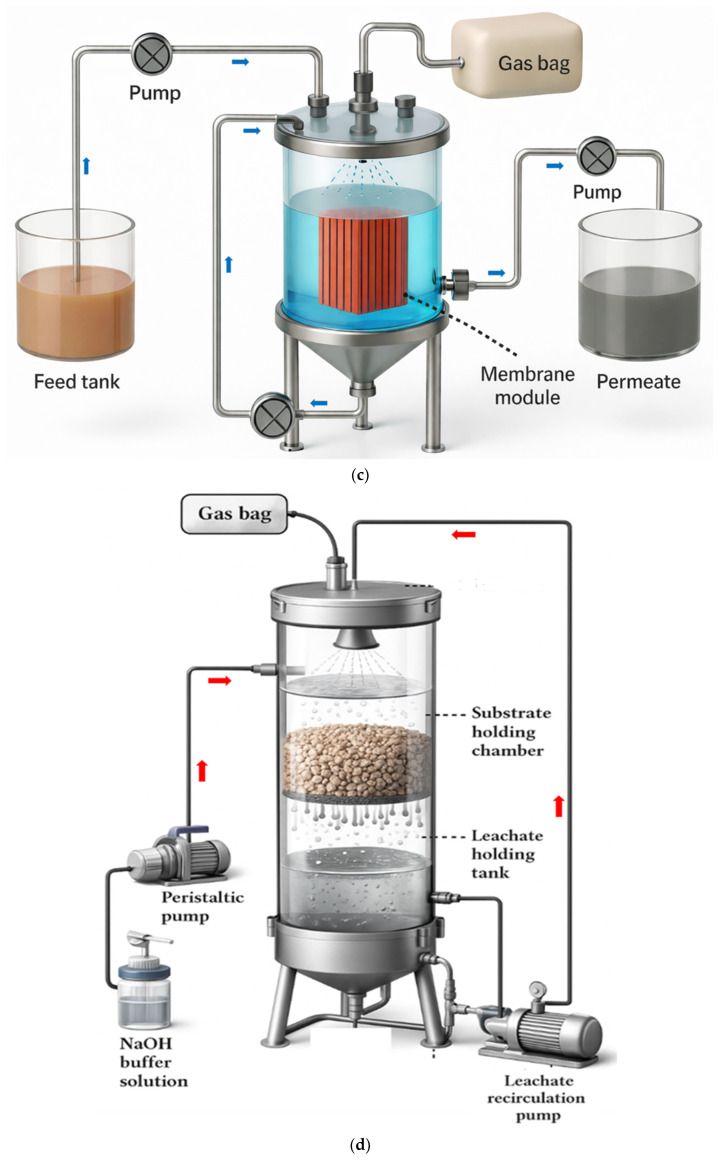
Schematic description of different reactor configurations used for acidogenic fermentation. (**a**) Continuous stirred tank reactor (CSTR); (**b**) up-flow anaerobic sludge blanket (UASB) reactor; (**c**) anaerobic membrane bioreactor (AnMBR); (**d**) leachate bed reactor (LBR).

Apart from substrate composition, reactor configuration plays a crucial role in influencing hydrolysis and acidogenesis efficiency. Different reactor configurations have been extensively studied for acidogenic fermentation, including the continuous stirred tank reactor (CSTR) [44,45], up-flow anaerobic sludge blanket (UASB) reactor [46,47], anaerobic membrane bioreactor (AnMBR) [48,49], and leachate bed reactor (LBR) [43,50] (Figure 2). These configurations, along with potential strategies that can be applied to overcome reactor-specific limitations and improve CA production, are discussed in the following sections.

### 3.1. Continuous Stirred Tank Reactor (CSTR)

**Figure 3:**
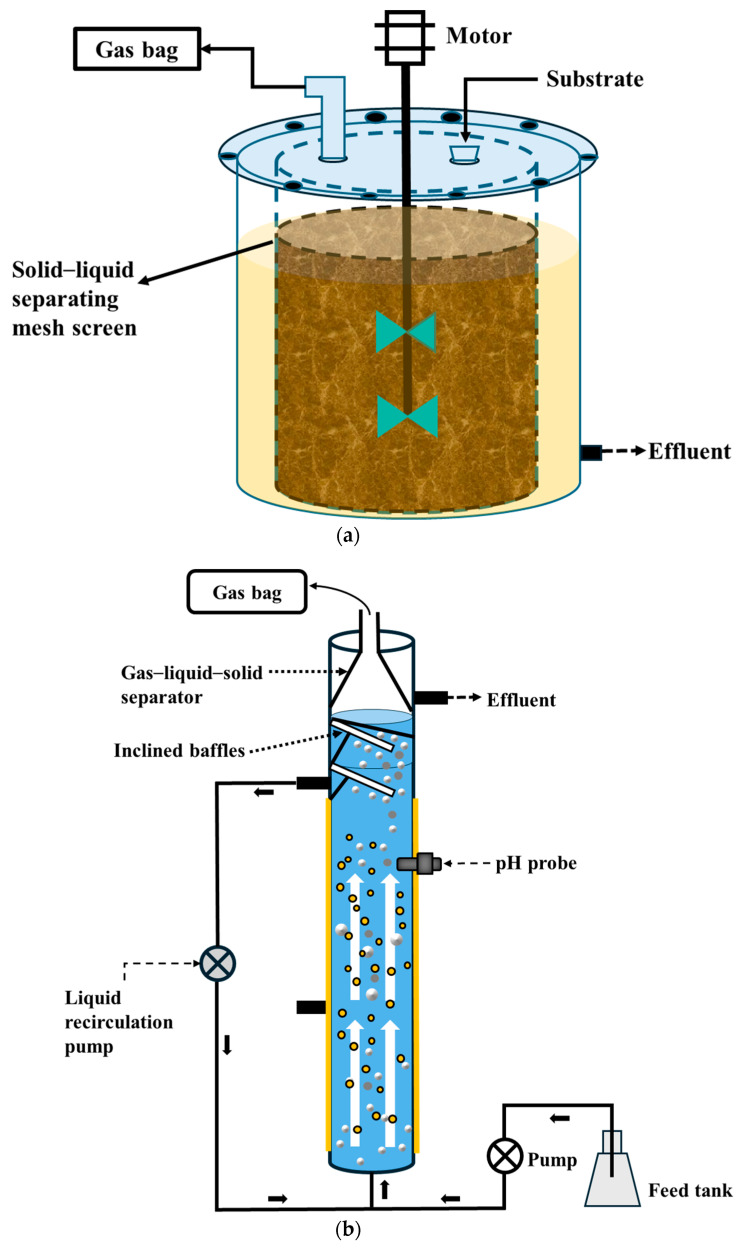

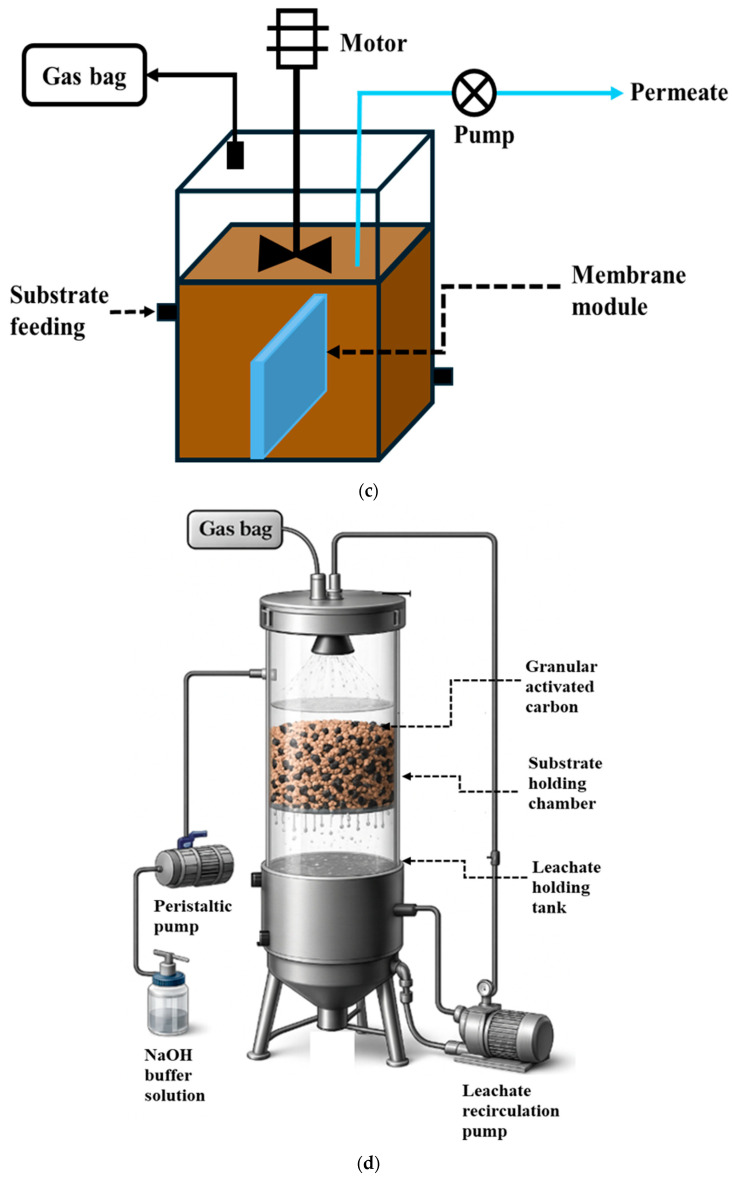

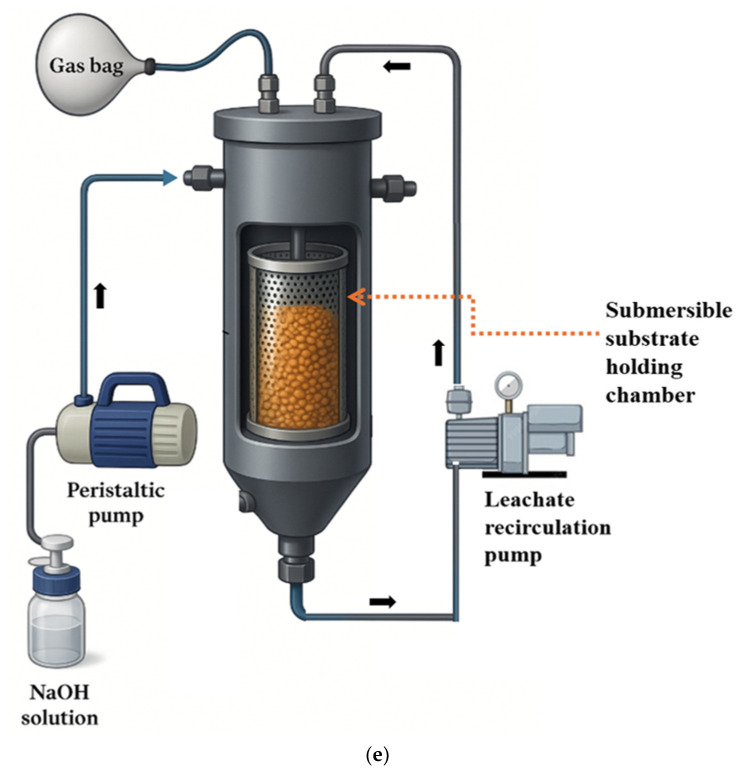
Design modifications to mitigate reactor-specific limitations and enhance CA production: (**a**) solid–liquid separating perforated mesh screen in CSTR; (**b**) expanded granular sludge bed (EGSB) configuration to improve hydrodynamic mixing and biomass–substrate contact; (**c**) anaerobic dynamic membrane bioreactor (AnDMBR) for in-situ solid–liquid separation with reduced membrane fouling; (**d**) granular activated carbon (GAC) supplementation to improve leachate permeability in LBR; and (**e**) submerging substrate holding chamber in leachate to improve mass transfer and prevent clogging in LBR.

CSTR is the most commonly used reactor type for acidogenic fermentation [44,51,52,53,54]. It contains impellers that provide continuous mixing, ensuring homogeneity of the reactor contents and improving contact between substrate and microorganisms, thereby enhancing CA production (Figure 2a) [53,55]. For instance, Greses et al. [53] operated a CSTR fed with carbohydrate- and protein-rich food waste and observed a maximum CA production of 27.3 g COD/L, primarily consisting of butyric and caproic acids, which accounted for 58.2% and 35.2% of the total CA, respectively. Similarly, Zheng et al. [56] operated a CSTR treating carbohydrate- and protein- rich food waste and achieved the highest CA production of 50.05 g COD/L, with butyric acid being the dominant organic acid, accounting for 30.17% of the total CA. Overall, most studies using CSTR have reported CA production ranging from 2.17 to 50.05 g COD/L, with CA compositions predominantly comprising acetic, butyric, and caproic acids (Table 2). Despite these promising results, several limitations hinder the large-scale implementation of CSTR for CA production. The major limitation is their inefficiency in handling substrates with high solids content, as they have been found effective only for substrates with volatile solids (VS) content up to 15%. As a result, this often necessitates dilution with water, resulting in larger reactor volume and operation at a lower organic loading rate. In addition, the continuous mixing makes CSTR energy-intensive, leading to higher operational costs [2,57,58]. Another main limitation is the risk of microbial biomass washout, which can hamper reactor performance [2,59]. These limitations, however, can be addressed through the potential strategies discussed below:

1. Microbial biomass washout in conventional CSTR can be mitigated by incorporating a cylindrical perforated mesh screen inside the reactor to enable solid–liquid separation. Karthikeyan et al. [60] developed a novel reactor configuration termed the solid–liquid separating CSTR (SLR-CSTR), in which a non-corrosive cylindrical perforated mesh screen (diameter: 6.5 cm, height: 28 cm, and pore size: 0.5 mm) was installed inside the reactor as a solid–liquid separator, as shown in Figure 3a. The reactor was loaded with carbohydrate- and protein-rich food waste (2 kg), anaerobic sludge (0.5 L), and water (1 L), and continuously mixed using a mechanical stirrer. With this reactor configuration, they successfully retained active biomass and unhydrolyzed solids, while achieving the maximum CA production of 25 g COD/L corresponding to an acidification degree of 89%. As a result, this design allowed for solids retention time (SRT) and hydraulic retention time (HRT) to be decoupled and prevented biomass washout.
2. In CSTR, mixing is essential to maintain uniform substrate distribution, prevent solid settling, and increase contact between microbes and substrates [61,62]. The reactor can be operated under two main mixing modes: continuous (at low or high mixing intensity) and intermittent. Low mixing intensity can cause accumulation of solids at the bottom of the reactor, formation of dead zones, and reduce mass transfer. On the other hand, high mixing intensity can generate strong shear forces that damage bacterial flocs, cause foam and scum formation, and increase energy consumption [58,61]. Both of these mixing intensities negatively affect hydrolysis and acidogenesis, ultimately leading to reactor failure. Intermittent mixing refers to a mode in which mixing is alternately turned on and off according to predetermined time intervals, ranging from seconds to hours [63]. Therefore, optimizing mixing mode is vital to maintain homogeneity, improve mass transfer, and minimize energy demand and operating costs. In this context, intermittent mixing is a promising approach to address these imitations and has already shown its effectiveness in improving biogas production in various studies [64,65,66]. Recently, Ma et al. [41] applied an intermittent mixing strategy in a CSTR fed with protein-rich sewage sludge and achieved the highest CA production of 3.88 g COD/L, which was significantly higher (*p* < 0.05) than that obtained under continuous mixing. Furthermore, intermittent mixing promoted the conversion of complex dissolved organic matter into CA and increased the relative abundance of *Tissierella* and *Bacillus*, both of which contributed to higher CA production.

**Table 2:** Summary of various reactor configurations applied for CA production.

Reactor Configuration	Substrate	Main Component of Substrate	Inoculum Source	Mode	Working Volume (L)	Temperature (°C)	Fermentation Time (d)	Organic Loading Rate	Mixing Speed (rpm)	pH	SCOD (g/L)	CA (g COD/L)	CA Composition	Main CA Produced	References
CSTR	Food waste	Carbohydrate & protein	Anaerobic sludge from WWTP and biogas plant	Semi-continuous	1	25	110	3 g VS/L.d	ng	6	86.2	27.3	Acetic acid: 3%	Butyric acid	[53]
													Propionic acid: 2%		
													Butyric acid: 58%		
													Valeric acid: 2%		
													Caproic acid: 35%		
	WAS & food waste	Protein & carbohydrate	Activated sludge from WWTP	Semi-continuous	4	55	28	8 g VS/L.d	ng	4.5–6	12	6	Acetic acid: 75%	Acetic	[52]
													Propionic acid: 8%		
													Butyric acid: 17%		
	Food waste	Carbohydrate & protein	Digested sludge from biogas plant	Semi-continuous	10	37	40	15 g VS/L.d	80	6	ng	50.05	ng	ng	[56]
	Pretreated macroalgae *Ulva*	Carbohydrate & protein	Anaerobic granular sludge from UASB reactor	Fed-batch	1.2	37	32	40 g VS/L	ng	7	ng	2.17	Acetic acid: 50%	Acetic and butyric	[67]
													Propionic acid: 5%		
													Butyric acid: 45%		
	Food waste	Carbohydrate & protein	Digested sludge from biogas plant	Semi-continuous	1	35	150	45 g VS/L.d	ng	Uncontrolled	ng	22.4	Acetic acid: 5%	-	[57]
													Propionic acid: 8%		
													* Lactic acid: 38%		
													* Ethanol: 49%		
	Kitchen waste	Carbohydrate & protein	Digested sludge from WWTP	Semi-continuous	8	37	30	5 g VS/L.d	100	7	ng	22.3	Acetic acid: 35%	Acetic and butyric	[62]
													Propionic acid: 21%		
													Butyric acid: 24%		
													Valeric acid: 3%		
													Caproic acid: 17%		
	Potato peel waste	Carbohydrate	AD sludge	Batch	5	37	6	71.5 g VS/L	300	Uncontrolled	24	18	Acetic acid: 37%	Butyric and acetic	[51]
													Propionic acid: 2%		
													Butyric acid: 61%		
	Potato peel waste	Carbohydrate	AD sludge	Batch	5	37	6	91.3 g VS/L	300	Uncontrolled	32	22	Acetic acid: 41%	Butyric and acetic	[51]
													Propionic acid: 9%		
													Butyric acid: 50%		
	Food waste & sewage sludge	Carbohydrate & protein	Sewage sludge from WWTP	Semi-continuous	200	55	60	4.8 kg VS/m^3^.d	ng	5.2	23	17	Acetic acid: 15%	Butyric	[54]
													Propionic acid: 7%		
													Butyric acid: 67%		
													Valeric acid: 5%		
													Caproic acid: 6%		
	Sewage sludge & municipal waste	Protein & carbohydrate	Anaerobic digested sludge from WWTP	Continuous	80	55	30	13.66 kg VS/m^3^.d	ng	9	ng	14.25	Acetic acid: 57%	Acetic and butyric	[44]
													Propionic acid: 12%		
													Butyric acid: 23%		
													Valeric acid: 8%		
UASB	Cheese whey	Carbohydrate	Acidogenic sludge from brewery WWTP	Continuous	1	30	60	15.1 g COD/L.d	0.16 ^d^	5	ng	3.66	Acetic acid: 47%	Acetic	[68]
													Propionic acid: 24%		
													Butyric acid: 12%		
													Valeric acid: 17%		
	Pretreated waste-activated sludge liquid	Protein	Anaerobic granular sludge from UASB reactor	Continuous	5	37	60	8 kg COD/m^3^.d	12 ^d^	Uncontrolled	ng	6.54	Acetic acid: 62%	Acetic	[69]
													Propionic acid: 21%		
													Butyric acid: 17%		
	Kraft foul condensate	Carbohydrate	Anaerobic granular sludge from UASB reactor	Continuous	0.5	55	25	8.6 g COD/L.d	ng	Uncontrolled	ng	3.14	Acetic acid: 54%	Acetic	[46]
													Propionic acid: 13%		
													Butyric acid: 22%		
													Valeric acid: 11%		
AnMBR	Apple pomace & potato protein liquor	Protein & carbohydrate	Rumen fluid	Semi-continuous	350	37	105	2 g VS/L.d	ng	Self-sustained 6–6.9	ng	10	Acetic acid: 21%	Butyric	[70]
													Propionic acid: 2%		
													Butyric acid: 42%		
													Valeric acid: 3%		
													Caproic acid: 32%		
	Kitchen waste slurry	Carbohydrate & protein	Digested sludge from biogas plant	Semi-continuous	45	38	120	15 kg COD/m^3^.d	ng	5	ng	34.4	Acetic acid: 38%	Butyric and acetic	[71]
													Propionic acid: 5%		
													Butyric acid: 42%		
													Valeric acid: 5%		
													Caproic acid: 10%		
	Kitchen waste slurry	Carbohydrate & protein	Digested sludge from biogas plant	Semi-continuous	45	38	120	15 kg COD/m^3^.d	ng	6	ng	42	Acetic acid: 50%	Acetic	[71]
													Propionic acid: 20%		
													Butyric acid: 15%		
													Valeric acid: 3%		
													Caproic acid: 12%		
	Cow manure	Carbohydrate & protein	Digested sludge from biogas plant	Semi-continuous	2	37	114	4.7 g VS/L.d	ng	Uncontrolled	17.5	6.93	Acetic acid: 65%	Acetic	[72]
													Propionic acid: 4%		
													Butyric acid: 16%		
													Valeric acid: 13%		
													Caproic acid: 2%		
	Excess sewage sludge	Protein	Granular sludge from UASB reactor	Semi-continuous	2	37	30	3 g VS/L.d	100	12	ng	9.8	Acetic acid: 54%	Acetic	[73]
													Propionic acid: 15%		
													Butyric acid: 15%		
													Valeric acid: 16%		
	Kitchen waste slurry	Carbohydrate & protein	Digested sludge from biogas plant	Semi-continuous	50	38	120	12.7 kg COD/m^3^.d	1 ^c^	9	ng	60.3	Acetic acid: 60%	Acetic	[74]
													Propionic acid: 24%		
													Butyric acid: 13%		
													Valeric acid: 3%		
	Food waste	Carbohydrate, protein & lipid	Digested sludge from AD reactor	Semi-continuous	2	37	34	10 g VS/L.d	150	5.5	ng	37	Acetic acid: 26%	Butyric	[48]
													Propionic acid: 9%		
													Butyric acid: 37%		
													Valeric acid: 15%		
													Caproic acid: 13%		
LBR	Food waste	Carbohydrate	Digested sludge from biogas plant	Semi-continuous	4	35	45	22 g VS/L.d	ng	7	ng	11.8	Acetic acid: 34%	Mixed CAs (near equal proportions)	[75]
													Propionic acid: 34%		
													Butyric acid: 32%		
	Food waste	Carbohydrate & protein	AD sludge	Batch	10	50	14	17 g VS/L	0.37 ^d^	7	82	36.5	Acetic acid: 27%	Butyric	[50]
													Propionic acid: 8%		
													Butyric acid: 57%		
													Valeric acid: 4%		
													Caproic acid: 4%		
	Food waste	Carbohydrate & protein	AD sludge	Batch	7.5	22	14	21.7 g VS/L	72 ^d^	6	33.33	28	Acetic acid: 25%	Butyric	[76]
													Propionic acid: 4%		
													Butyric acid: 71%		
	Vegetable waste	Carbohydrate	Anaerobic sludge from WWTP	Batch	5	35	10	6.7 g VS/L	ng	8	29.1	27.91	Acetic acid: 55%	Acetic	[20]
													Propionic acid: 3%		
													Butyric acid: 34%		
													Valeric acid: 8%		
	Food waste	Carbohydrate & protein	Anaerobic sludge from WWTP	Batch	12	22	14	17.3 g VS/L	72 ^d^	7	ng	18	Acetic acid: 35%	Butyric	[77]
													Propionic acid: 16%		
													Butyric acid: 46%		
													Valeric acid: 3%		
	Food waste	Carbohydrate & protein	Anaerobic sludge from WWTP	Batch	7.5	22	14	21.7 g VS/L	72 ^d^	6	33	24	Acetic acid: 29%	Butyric	[78]
													Propionic acid: 4%		
													Butyric acid: 67%		
	Food waste	Carbohydrate & protein	Anaerobic sludge from WWTP	Batch	7.5	22	14	21.7 g VS/L	72 ^d^	8	35	27	Acetic acid: 52%	Acetic	[78]
													Propionic acid: 18%		
													Butyric acid: 30%		

AD—anaerobic digestion; WWTP—Wastewater treatment plant; WAS—waste-activated sludge; VS—volatile solids; SCOD—soluble chemical oxygen demand; CSTR—continuous stirred tank reactor; UASB—up-flow anaerobic sludge blanket; AnMBR—anaerobic membrane bioreactor; LBR—leachate bed reactor; ng—not given. ^c^ Recirculation in m^3^/h; ^d^ Recirculation in L/h; * dominant fermentation products.

### 3.2. Up-Flow Anaerobic Sludge Blanket (UASB) Reactor

High-rate anaerobic digesters, such as up-flow anaerobic sludge blanket (UASB) reactors, are widely used for the treatment of both low- and high-strength wastewater [79]. It consists of three main components: the sludge bed, the sludge blanket, and the gas–liquid–solid separator (Figure 2b). It operates based on the up-flow movement of wastewater from the bottom of the reactor through the sludge blanket containing active granules, where organic matter is degraded and converted into CA [46,80]. Calero et al. [68] operated the UASB reactor fed with carbohydrate-abundant cheese whey at different organic loading rates and observed the highest CA production of 3.66 g COD/L, corresponding to a 97% degree of acidification at an organic loading rate of 15.1 g COD/L.d. Interestingly, Zhang et al. [69] employed a UASB reactor to simultaneously produce CA and recover nutrients (nitrogen and phosphorus) from the liquid fraction of pretreated protein-dominant waste-activated sludge. They achieved a maximum CA concentration of 6.54 g COD/L and a struvite recovery efficiency of 1.98 g/g PO_4_^3−^. Despite these promising results, the USAB reactor faces several limitations when applied to CA production. Firstly, it is highly sensitive to variation in organic loading rate, which frequently leads to process instability and unintended carbon conversion towards biogas rather than CAs [4,46]. Secondly, the slow start-up and dense granule formation limit the contact between the substrate and microbial biomass, thereby reducing substrate hydrolysis, especially when treating complex substrates. Finally, the high risk of microbial biomass washout when treating substrates with high suspended solids content, the inhibition resulting from product accumulation, and poor phase separation can further impact reactor performance [80]. These limitations can be addressed using the strategies discussed below:

1. Increasing up-flow velocity in the anaerobic sludge bed reactor can effectively address several limitations associated with the conventional UASB reactor [80]. Operating the reactor at higher hydraulic velocity improves hydrodynamic mixing and increases contact between biomass and wastewater, resulting in better substrate utilization [80,81]. For instance, Archilla et al. [82] operated an expanded granular sludge bed (EGSB) reactor (as shown in Figure 3b), which is an advanced configuration derived from the UASB concept, using leachate derived from the thermally diluted acid hydrolysis of carbohydrate-abundant BSG and reported the highest CA production of 120 mmol/L at 24 HRT, corresponding to an acidification level of 83%.
2. Incorporating packing materials into the UASB reactor can improve process performance by retaining microbial biomass and providing a larger surface area for biofilm development [80]. This improves contact between biomass and substrate, thereby accelerating substrate degradation and increasing CA production. Various packing materials used for biogas production and CO_2_ biomethanation have shown promising results. These materials can be categorized into three main categories: (i) natural packing materials, such as vermiculite shales, loofah, and crushed clay; (ii) commercial packing materials, such as Raschig rings, Hel-X, polyurethane foam, glass rings, and Hiflow rings; and (iii) carbonaceous materials, such as biochar, pyrochar, magnetite, and graphene [58,81]. For instance, Kougias et al. [83] investigated the effect of packing materials in up-flow reactors fed with biogas for (methane) CH_4_ upgrading and found that the reactor containing packing material achieved an 81% CH_4_ content, compared to only 60% CH_4_ content in the reactor without packing material. Similarly, Wambugu et al. [84] operated a biochar-amended UASB reactor treating diluted food waste and reported a 77% increase in biogas yield compared to a reactor operated without the biochar addition. However, the use of these packing materials in a UASB reactor, especially for CA production, has yet to be reported. Therefore, future research focused on investigating the feasibility and effectiveness of incorporating packing materials in the UASB reactor for CA production is worth exploring.
3. The accumulation of CA production in the reactor is detrimental to microorganisms because undissociated CAs can penetrate the cell membrane and disrupt microbial processes. Furthermore, the CA accumulation leads to a drop in pH, which inhibits intracellular enzyme activities and consequently impedes acidogenic fermentation [85]. Therefore, the timely extraction of CAs from the fermentation broth is essential to maintain stable reactor performance and enhance CA production [4,85]. Various conventional methods for CA recovery have been studied, including crystallization, precipitation, distillation, and solvent extraction [4,86]. However, these methods are energy-intensive and expensive, accounting for around 35% of the total process cost [47,86]. As a result, there is growing interest in in situ CA recovery methods, such as electrodialysis, gas stripping, membrane separation, and electrochemical cells systems. These recovery methods enable continuous CA separation directly from the reactor without interrupting the fermentation process, unlike conventional methods where CAs are first produced and then recovered [4,86]. Recently, Castilla-Archilla et al. [47] demonstrated the successful coupling of electrochemical cells with a UASB reactor for in-situ CA recovery from fermentation broth treating carbohydrate-rich glucose and achieved the highest CA recovery of 29.09 g COD/L.

### 3.3. Anaerobic Membrane Bioreactor (AnMBR)

Anaerobic membrane bioreactor (AnMBR) integrates acidogenic fermentation and membrane filtration, with the membrane submerged in the reactor (Figure 2c) [4,74]. This configuration decouples the HRT from the SRT, enabling prolonged microbial biomass retention and preventing microbial washout [74,85]. Furthermore, the membrane facilitates the continuous separation of soluble metabolites from the fermentation broth, thereby mitigating product inhibition, promoting substrate conversion efficiency, and improving overall productivity [73,85]. The AnMBR has been employed for the treatment of various substrates such as wastewater, food waste, sewage sludge, kitchen waste slurry, and animal manure for VFA production [4,71,72,73,85]. For instance, Xiao et al. [71] achieved a maximum CA production of 42 g COD/L with an acidification level of 80% during the fermentation of carbohydrate- and protein-rich kitchen waste slurry in a pilot-scale AnMBR. Likewise, Parchami et al. [70] operated a pilot-scale AnMBR fed with protein- and carbohydrate-rich apple pomace and potato protein liquor and reported stable CA production of 10 g COD/L with a CA yield of 470 g COD_CA_/kgVS_added_ over 105 days of operation. In general, studies conducted using AnMBR have reported CA production ranging from 6.93 g COD/L to 60.30 g COD/L (Table 2) [48,72,73,74]. These recent advancements in acidogenic fermentation for CA production using AnMBR currently correspond to a technology readiness level (TRL) of 5, progressing toward TRL 6, which represents the demonstration of technology in a relevant environment. However, several challenges remain in pilot-scale AnMBR, including membrane fouling and high operational cost, which hinder scalability to full-scale applications. Therefore, further studies are required to address these limitations to facilitate industrial-scale implementation. Potential strategies to overcome these challenges are discussed as follows:

1. The anaerobic dynamic membrane bioreactor (AnDMBR) is an anaerobic membrane system that operates on a similar principle to conventional AnMBR, but it incorporates the in-situ formation of a biological cake layer, referred to as the dynamic membrane, on the surface of a support material, as shown in Figure 3c. This dynamic membrane provides effective filtration and addresses several limitations associated with AnMBR [87,88]. Materials such as stainless steel meshes and fabrics with macropores are commonly used as support surfaces to facilitate the formation of the dynamic membrane [87,89]. Once the dynamic membrane becomes fully fouled, the cake layer can be easily removed, cleaned, and replaced by a newly formed layer [87]. Furthermore, dynamic membranes have a smaller pore size than the supporting material, thereby enhancing filtration performance and producing permeate with total suspended solids concentrations below 10 mg/L [89]. Lower operational cost, higher membrane flux, and ease of fouling control are the major advantages of the AnDMBR [89,90]. Liu et al. [91] utilized the supernatant derived from thermally pretreated protein-abundant sewage sludge in an AnDMBR and achieved a maximum CA yield of 380 g COD_CA_/kgVS_added_ and a CA production of 60 g COD/L. The dynamic membrane remained stable for 70 days, during which the membrane flux increased from 6.25 to 25 L/m^2^.d. Likewise, Fonoll et al. [92] operated an AnDMBR fed with carbohydrate- and protein-rich food waste for 110 days without any disruption and achieved a CA yield of 550 g COD_CA_/kgVS_added_ along with removal rates of 58.9% and 69% for neutral detergent fiber and acid detergent fiber, respectively, present in the food waste.
2. Reduction in operational costs and improvement in reactor performance can be achieved by integrating AnMBR with a forward osmosis (FO) membrane [87]. FO allows for water to pass through a semi-permeable membrane from a low-osmotic-pressure feed solution to a high-osmotic-pressure draw solution [87]. Integrating FO with AnMBR significantly reduces energy consumption by eliminating the need for energy-intensive filtration processes compared to conventional AnMBR [87]. Chen et al. [93] operated an AnMBR coupled with a two-stage FO process to simultaneously recover organic matter in the form of CAs, nitrogen, and phosphorus from low-strength protein-dominant municipal wastewater. In this system, the first FO membrane was used to concentrate municipal wastewater, which was then fed into the AnMBR for CA production. Subsequently, the AnMBR effluent was further concentrated using a second FO membrane to recover ammonia nitrogen (NH_4_^+^-N) and total phosphorus (TP) through the struvite precipitation method, while residual CAs were directly recovered. With this reactor configuration, the first FO membrane concentrated the municipal wastewater to 1/10th of the initial volume, increasing the concentrations of COD, NH_4_^+^-N, and TP to 2.80 g/L, 0.20 g/L, and 0.03 g/L, respectively. Feeding this concentrated wastewater into the AnMBR resulted in a CA production of 1.78 g COD/L, primarily consisting of acetic and propionic acids. The subsequent FO stage further concentrated NH_4_^+^-N and TP in the AnMBR effluent to 0.17 g/L and 0.04 g/L, respectively. The struvite precipitation method led to recovery efficiencies of 94.53% for NH_4_^+^-N, and 98.59% for TP, while the CA concentration of 2.9 g COD/L was obtained from the residual solution. Although the CA concentration was relatively lower due to the use of low-strength municipal wastewater, this reactor configuration demonstrates a promising approach for the simultaneous recovery of organic matter (in the form of CAs) and nutrients. Therefore, further research is needed to enhance the overall resource recovery efficiency from the integrated system.

### 3.4. Leachate Bed Reactor (LBR)

In comparison to other reactors, dry fermenters such as the leachate bed reactor (LBR) offer distinct advantages for CA production. These include the ability to accommodate waste with a high solids content (~30–40%) and operate without the need for substrate dilution with process water or continuous mixing, resulting in lower operational costs and energy savings [2,43]. It consists of a substrate holding chamber with a perforated base, separated from the leachate holding tank positioned at the bottom of the reactor (Figure 2d). The leachate that drains into the tank is periodically recirculated and sprayed over the substrate from the top. This intermittent recirculation removes the need for continuous mixing while achieving relatively higher hydrolysis and CA production [2]. The majority of the acidogenic fermentation studies carried out in the LBR reported hydrolysis yields ranging from 227 to 751 g SCOD/kgVS_added_ and CA yields ranging from 37 to 621 g COD_CA_/kgVS_added_ [2,20,50,76,78]. However, substrate bed clogging and mass transfer limitations have been recognized as major challenges that need to be addressed to promote CA production in LBRs [2]. These challenges can be overcome using the strategies discussed as follows:

1. Substrate bed clogging in the LBR can be mitigated by adding materials that help maintain bed permeability. These include organic materials (e.g., woodchips and sawdust), inorganic materials (e.g., plastic particles and plastic hollow spheres), and carbonaceous materials (e.g., granular activated carbon, GAC) in the substrate holding chamber [43,94]. Recently, Radadiya et al. [43] operated an LBR fed with carbohydrate- and protein-abundant food waste supplemented with GAC (Figure 3d) at a loading of 0.51 g GAC/gVS_food waste_ and achieved a VFA yield of 507 g COD_CA_/kgVS_added_, which was 35% higher than that of an LBR without GAC loading (375 g COD_CA_/kgVS_added_).
2. Theoretically, gas–liquid mass transfer limitations in the LBR can be alleviated by increasing the gas–liquid mass transfer coefficient and mass transfer driving force, both of which are influenced by factors such as stirring and pressure [58]. Since stirring is absent in the LBR, controlling pressure becomes an effective way to overcome mass transfer limitations. The LBR can be operated under pressurized conditions by applying either exogenous pressure (e.g., flushing headspace with external gases) or endogenous pressure (e.g., autogenerated pressure resulting from the accumulation of H_2_ and CO_2_ produced during acidogenic fermentation) [42,95,96]. Various previous studies reported 24–61% improvement in CA production when the reactor was operated under pressurized conditions during acidogenic fermentation [96,97,98]. Recently, Luo et al. [99] investigated the effect of autogenerated pressure in an LBR fed with carbohydrate- and protein-rich food waste and reported a CA yield of 407 g COD_CA_/kgVS_added_, which was significantly higher (*p* < 0.05) than that of an LBR operated without autogenerated pressure (365 g COD_CA_/kgVS_added_).
3. Submerging the substrate holding chamber in the leachate, combined with intermittent leachate recirculation, can help overcome mass transfer limitations and substrate bed clogging in the LBR [59]. This configuration enhances contact between the substrate and microorganisms, improves diffusion of soluble organics and metabolites, and increases enzyme accessibility for substrate hydrolysis. Furthermore, the intermittent leachate recirculation maintains a uniform distribution of nutrients and prevents preferential flow channeling within the reactor [2]. Recently, Singh et al. [59] applied this approach (Figure 3e) and obtained a high hydrolysis yield of 628 g SCOD/kgVS_added_ and a CA yield of 517 g COD_CA_/kgVS_added_ from carbohydrate- and protein-rich food waste at a high volumetric organic loading of 55 gVS/L_reactor_ without any operational issues. These promising results indicate that future studies should explore the applicability of this strategy for the bioconversion of various organic wastes into CAs.

## 4. Inoculum Selection

An efficient and robust acidogenic fermentation depends on the inoculum containing functional hydrolytic and acidogenic bacteria [40,43,86]. These microbial consortia govern substrate hydrolysis and the subsequent conversion of soluble monomers into CAs, thereby determining process efficiency. The most common hydrolytic bacteria belong to the genera of *Bacteroides*, *Streptococcus*, *Enterobacter*, *Lactobacillus*, *Bifidobacterium*, and *Sphingomonas* [1]. In contrast, the dominant acidogenic bacteria are primarily associated with the genera of *Clostridium*, *Enterobacter*, *Prevotella*, *Bifidobacterium*, *Pseudomonas*, and *Bacillus* [1]. An imbalance between these bacterial groups can limit the fermentation reactions, resulting in lower CA production [100]. In this context, inocula are classified into aerobic and anaerobic inocula, enriched inoculum, and rumen microorganisms, which are discussed in the following subsections.

### 4.1. Aerobic and Anaerobic Inocula

Aerobic inoculum contains microorganisms that require oxygen, whereas anaerobic inoculum consists of microorganisms that grow in the absence of oxygen. Anaerobic sludge, granular sludge, and animal manure obtained from biogas plants and livestock farms are commonly used as anaerobic inoculum. These inocula have demonstrated effectiveness in achieving higher CA yields, primarily due to the greater relative abundance of fermentative microorganisms like *Prevotella*, *Bacteroides*, *Bifidobacterium*, *Corynebacterium*, and *Enterococcus*. The reported CA yields using anaerobic inoculum range from 205 g COD_CA_/kgVS_added_ to 669 g COD_CA_/kgVS_added_ [43,76,101,102]. WAS collected from wastewater treatment plants is typically used as an aerobic inoculum and contains a diverse consortium of hydrolytic and facultative microorganisms. These microbes are robust and tolerant to environmental fluctuations, making them easy to handle in both laboratory and industrial settings. Under anaerobic conditions, strict aerobic microorganisms become inactive, while facultative microorganisms shift their metabolic pathways toward fermentation, converting soluble monomers released by hydrolytic microorganisms into CAs [103]. However, because the microbial community in aerobic inoculum is not specialized for acidogenic fermentation, CA production is generally lower than that obtained using anaerobic inoculum. Several studies have reported increased CA production from carbohydrate- and protein-rich substrates such as olive mill wastewater, rice straw, and food waste using aerobic inoculum [104,105,106]. This improvement is mainly attributed to the higher abundance of both hydrolytic and facultative bacteria in the aerobic inoculum, such as *Clostridium pasteurianum*, *Clostridium stercorarium*, and *Thermoanaerobacterium saccharolyticum*, which facilitates substrate degradation and enhances acidogenic efficiency [43,106,107]. In contrast, Jodhani et al. [108] reported a lower CA yield of 131 g COD_CA_/kgVS_added_ from carbohydrate- and protein-rich food waste using WAS compared to 182 g COD_CA_/kgVS_added_ obtained with anaerobic sludge. Both anaerobic and aerobic inocula can improve CA production, with the former being generally employed due to their superior performance. Overall, anaerobic inoculum generally requires longer adaptation periods and exhibits slower start-up compared to aerobic inoculum due to slower microbial growth and energy-limited metabolism. However, once adapted, they are more stable and produce higher CA production. On the contrary, the aerobic inoculum adapts more rapidly because of faster growth rates, but it results in lower CA production under anaerobic conditions, as most aerobic microorganisms become metabolically inactive or decay in the absence of oxygen, thereby reducing CA production [109,110].

### 4.2. Enriched Inoculum

Enriched inoculum serves as a promising source of functionally active microbes that can rapidly acclimatize to reactor operating conditions, thereby increasing hydrolysis and CA production within a shorter fermentation time [43]. This enriched inoculum can be obtained either from a continuously operated reactor or at the end of reactor operation during acidogenic fermentation. For example, Radadiya et al. [43] used an enriched inoculum with a high relative abundance of *Prevotella* (30%), *Bacteroides* (30%), *Enterococcus* (13%), *Clostridium* (5%), and *Solobacteria* (4.8%) compared to a non-enriched inoculum mostly dominated by *Enterococcus* (20%), *Bacteroides* (14%), and *Prevotella* (12%). The enriched inoculum, obtained after 14 days of LBR operation, resulted in a 10–22% increase in hydrolysis and CA yields from carbohydrate- and protein-dominant food waste, achieving 683 g SCOD/kgVS_added_ and 617 g COD_CA_/kgVS_added_, respectively. Similarly, Shewa et al. [77] evaluated the LBR performance using two different inoculums: anaerobic sludge and enriched inoculum (containing a mixture (1:1) of enriched microbial biomass and anaerobic sludge). The authors observed 36% and 51% higher hydrolysis and CA yields, respectively, with the enriched inoculum, which contained a high relative abundance of *Roseburia* (52%) and *Eubacterium* (10%), compared to the anaerobic sludge, which was mostly dominated by *Bacteroides* (15%) and *Pseudomonas* (10%). Likewise, in another study, a high hydrolysis yield of 774 g SCOD/kgVS_added_ and a CA yield of 697 g COD_CA_/kgVS_added_ were obtained by using enriched inoculum at a fermentation time of 10 days [111]. The significant improvement in the performance of acidogenic fermentation using enriched inoculum can be attributed to increased relative abundance of acclimated hydrolytic and acidogenic bacteria, which enhance the reactor system’s robustness and efficiency. However, despite these advantages, inoculum enrichment is inherently time-intensive, as the selective enrichment and acclimation of microbial consortia require a prolonged incubation period for stable reactor operation.

### 4.3. Rumen Microorganisms

Rumen microorganisms, such as bacteria, fungi, and protozoa present in the alimentary canal of ruminant animals, have demonstrated high efficiency in converting lignocellulosic biomass into CA [21,112,113]. For instance, Liang et al. [114] reported a maximum CA production of 10.2 g COD/L, along with hemicellulose and lignin degradation efficiencies of 55.2% and 7.01%, respectively, from protein-rich corn straw using rumen fluid containing the dominant bacterial genera *Succiniclasticum* and *Treponema*, and the fungal genus *Neocallimastix*. Interestingly, Nguyen et al. [22] developed a novel rumen–AnMBR system equipped with a submerged hollow fiber membrane module, using rumen fluid as the inoculum, dominated by *Ruminococcaceae* (13.3%) and *Prevotella* (7.5%), and carbohydrate-rich maize silage as the substrate, achieving a CA yield of 0.44 g COD_CA_/kgVS_added_ under continuous operation. Similar improvements in CA production have been demonstrated in other studies utilizing rumen microbes as inoculum [115,116,117,118]. However, their metabolic activity often declines during long-term operation, and the genetic and metabolic mechanisms responsible for their hydrolytic and acidogenic performance remain largely uncharacterized [113]. These limitations currently hinder their commercial application, despite their promising potential.

## 5. Challenges and Future Perspectives

Acidogenic fermentation is a promising biotechnology for converting organic wastes into value-added CAs. However, several key challenges, such as low substrate hydrolysis and reactor-specific limitations that consequently lead to low CA production, need to be addressed. These challenges and perspectives are discussed as follows:

1. Hydrolysis is the rate-limiting step in acidogenic fermentation because of the substrate’s rigid and complex structure [1]. The hydrolysis products are essential components needed for CA production. In this regard, various biological pretreatment methods, such as enzymatic, fungi, and bacterial, can be applied to improve substrate hydrolysis and CA production [1]. These biological pretreatments rely on the activity of microorganisms or enzymes under mild operational conditions to break down the complex structure of substrate without generating inhibitory compounds, thereby reducing energy demand and costs for downstream processing [1]. For example, X. Yang et al. [119] pre-treated carbohydrate- and protein-rich kitchen waste with an enzymatic cocktail (cellulase and hemicellulase) and obtained a maximum CA production of 50.73 g COD/L, which was 63% higher than that obtained from unpretreated kitchen waste. Future studies should focus on investigating the feasibility of applying these biological pretreatments to substrates rich in carbohydrates, proteins, and lipids to enhance hydrolysis and acidogenesis efficiency.
2. Although several studies have reported that operational parameters such as pH, organic loading rate, and temperature affect acidogenesis, the influence of substrate composition has often been overlooked [2,4,9]. The substrate composition, mainly the proportions of carbohydrates, proteins, and lipids, strongly influences CA production and composition, in spite of the concurrent effects of operational parameters, as illustrated in Table 1 [8]. Therefore, future research should place greater emphasis on substrate composition by systematically characterizing and quantifying carbohydrate, protein, and lipid contents, elucidating associated metabolic pathways, and evaluating their impacts on hydrolysis and acidogenesis processes.
3. Reactor configurations such as CSTR, UASB, AnMBR, and LBR have been extensively studied for acidogenic fermentation. Although various potential strategies have been proposed to address reactor-specific limitations and enhance CA production, their applicability remains mostly limited to the laboratory scale. Future research should focus on validating these strategies in pilot-scale studies and scaling them up for full-scale implementation. Furthermore, integrating different reactor configurations, such as LBR-AnMBR and CSTR-UASB, could provide synergistic benefits by enabling the treatment of various substrates, improving mass transfer, increasing substrate degradation, and enhancing CA production. Likewise, coupling acidogenic fermentation with in situ CA recovery techniques, such as electrodialysis, membrane separation, and electrochemical cell-assisted systems, could enable continuous CA production and removal, thereby maintaining stable reactor performance during long-term operation.
4. Co-fermentation, which involves the simultaneous digestion of two or more substrates, has the potential to enhance CA production by providing additional carbon sources and essential nutrients for microbial growth [1,38]. For instance, Feng et al. [120] achieved a CA yield of 0.59 gSCOD/gVS from the co-fermentation of carbohydrate- and protein-abundant mushroom champost and sewage sludge, which was around 37% and 14% greater than fermentation of champost and sludge, respectively. However, further studies are required to determine the optimum substrate mixing ratio and identify suitable complementary substrates for co-fermentation.
5. An efficient and robust acidogenic fermentation depends on the inoculum, as it contains functional hydrolytic and acidogenic bacteria [43,86]. An imbalance between these bacterial groups can hinder the fermentation processes, resulting in lower SCOD and CA production. In this context, employing enriched inocula that are functionally active and rapidly acclimatize to reactor operating conditions could be a promising strategy to improve hydrolysis and CA production within a shorter fermentation time [43]. The positive outcomes of the enriched inoculum have already been demonstrated in LBRs treating carbohydrate- and protein-dominant food waste at very high organic loading rates [43,59]. Thus, future research should investigate the use of enriched inoculum for treating various organic wastes in different reactor configurations and evaluate their performance in terms of hydrolysis and CA production. Similarly, rumen microorganisms represent an excellent source of hydrolytic bacteria for degrading recalcitrant components of lignocellulosic biomass. Most studies using rumen microbes for CA production have been conducted in AnMBR, but continuous operation remains limited, and the method is still in its infancy [113]. Hence, future research should focus on developing rumen-driven AnMBR for the treatment of diverse organic wastes to facilitate continuous CA production and removal.

## 6. Conclusions

This review provides a comprehensive and up-to-date overview of substrate composition, inoculum selection, and recent advances in reactor configurations, along with strategies to address reactor-specific limitations and enhance CA production. Substrate composition in terms of carbohydrates, proteins, and lipids strongly influences both CA production and composition, yet it has often been overlooked. Compared to protein and lipid-rich substrates, carbohydrate-rich substrates generally yield higher CA production due to their greater biodegradability. These substrates have been widely utilized in different reactor configurations, demonstrating significant potential for CA production. However, several challenges, such as limited substrate hydrolysis, reactor-specific limitations, and lower CA production, still hinder progress towards full-scale implementation. To overcome these limitations, several potential strategies are recommended, including biological pretreatment methods, co-fermentation, reactor-specific techniques, and the use of enriched inoculum or rumen-derived microorganisms.

## Figures and Tables

**Table 1:** CA yield and composition obtained from different substrates.

Substrate	Main Component of Substrate	Inoculum Source	CA (g COD/L)	CA Productivity (g COD/L/d)	CA Yield	CA Composition	Main CA Produced	References
Glucose	Carbohydrate	Anaerobic granular sludge from UASB reactor	2.95	0.59	0.97 gCOD_CA_/g SCOD	Acetic acid: 37%	Butyric and acetic	[10]
						Propionic acid: 3%		
						Butyric acid: 60%		
Cheese whey permeate	Carbohydrate	Acidogenic biomass	2.27	0.13	0.60 gCOD_CA_/g SCOD	Acetic acid: 43%	Acetic and butyric	[11]
						Propionic acid: 15%		
						Butyric acid: 42%		
Papermill effluent	Carbohydrate	Acidogenic biomass	3.96	0.23	0.59 gCOD_CA_/g SCOD	Acetic acid: 9%	Butyric	[11]
						Propionic acid: 13%		
						Butyric acid: 78%		
Maize silage	Carbohydrate	Anaerobic sludge from biogas plant	ng	ng	0.78 gCOD_CA_/g SCOD	Acetic acid: 77%	Acetic	[12]
						Propionic acid: 10%		
						Butyric acid: 9%		
						Valeric acid: 4%		
Cheese whey	Carbohydrate	Anaerobic sludge from biogas plant	ng	ng	0.71 gCOD_CA_/g SCOD	Acetic acid: 82%	Acetic	[12]
						Propionic acid: 4%		
						Butyric acid: 8%		
						Valeric acid: 6%		
BSG	Carbohydrate	Anaerobic granular sludge from UASB reactor	16.9	5.63	ng	Acetic acid: 40%	Butyric and acetic	[16]
						Propionic acid: 1%		
						Butyric acid: 55%		
BSG	Carbohydrate	Anaerobic sludge from WWTP	35.5	2.21	ng	Acetic acid: 28%	Propionic	[17]
						Propionic acid: 41%		
						Butyric acid: 18%		
						Valeric acid: 13%		
Corn stover	Carbohydrate	AD sludge	10.53	ng	ng	Acetic acid: 94%	Acetic	[19]
						Propionic acid: 1%		
						Butyric acid: 4%		
						Valeric acid: 1%		
Potato peels	Carbohydrate	Anaerobic sludge from WWTP	27.92	4.65	0.46 gCOD_CA_/g VS	Acetic acid: 53%	Acetic and butyric	[20]
						Propionic acid: 13%		
						Butyric acid: 34%		
Corn stover	Carbohydrate	Rumen fluid	32.61	0.52	ng	Acetic acid: 67%	Acetic	[18]
						Propionic acid: 22%		
						Butyric acid: 9%		
						Valeric acid: 2%		
Corn stover	Carbohydrate	Rumen fluid	8.48	0.07	ng	Acetic acid: 58%	Acetic	[21]
						Propionic acid: 32%		
						Butyric acid: 10%		
Maize silage	Carbohydrate	Rumen fluid	ng	ng	0.44 gCOD_CA_/g VS	Acetic acid: 59%	Acetic	[22]
						Propionic acid: 25%		
						Butyric acid: 16%		
Potato peels	Carbohydrate	AD sludge	16	3.33	0.45 gCOD_CA_/g VS	Acetic acid: 30%	Propionic, acetic, and butyric	[13]
						Propionic acid: 32%		
						Butyric acid: 27%		
						Valeric acid: 6%		
						Hexanoic acid: 5%		
Slaughterhouse wastewater	Protein	AD sludge	1.50	0.15	0.35 gCOD_CA_/g SCOD	Acetic acid: 74%	Acetic	[23]
						Propionic acid: 3%		
						Butyric acid: 10%		
						Valeric acid: 13%		
Sewage sludge	Protein	AD sludge	4	0.40	0.33 gCOD_CA_/g SCOD	Acetic acid: 62%	Acetic	[23]
						Propionic acid: 10%		
						Butyric acid: 16%		
						Valeric acid: 12%		
Meat and bone meal	Protein	AD sludge	8	0.80	0.46 gCOD_CA_/g SCOD	Acetic acid: 50%	Acetic	[23]
						Propionic acid: 15%		
						Butyric acid: 13%		
						Valeric acid: 22%		
Tuna waste	Protein	AD sludge	25	0.62	0.73 gCOD_CA_/g SCOD	Acetic acid: 52%	Acetic	[24]
						Propionic acid: 11%		
						Butyric acid: 34%		
						Valeric acid: 3%		
Egg white	Protein	Anaerobic granular sludge from UASB reactor	7	0.28	0.26 gCOD_CA_/g VS	Acetic acid: 25%	Mixed CAs (equal proportions)	[25]
						Propionic acid: 25%		
						Butyric acid: 25%		
						Valeric acid: 25%		
Tofu	Protein	Anaerobic granular sludge from UASB reactor	12	0.48	0.46 gCOD_CA_/g VS	Acetic acid: 56%	Acetic	[25]
						Propionic acid: 16%		
						Butyric acid: 10%		
						Valeric acid: 18%		
Crude glycerol	Lipid	AD sludge	2.1	0.21	0.22 gCOD_CA_/g SCOD	Acetic acid: 15%	Propionic	[23]
						Propionic acid: 74%		
						Butyric acid: 6%		
						Valeric acid: 5%		
Olive mill solid waste	Lipid	AD sludge	3.69	0.09	ng	Acetic acid: 60%	Acetic	[26]
						Propionic acid: 23%		
						Butyric acid: 17%		
Gelatin-rich wastewater	Lipid	AD sludge	1.56	0.04	ng	Acetic acid: 35%	Acetic and butyric	[27]
						Propionic acid: 10%		
						Butyric acid: 30%		
						Valeric acid: 19%		
						Caproic acid: 6%		
Olive oil mill effluent	Lipid	AD sludge	10.7	0.35	0.28 gCOD_CA_/g SCOD	Acetic acid: 62%	Acetic	[28]
						Propionic acid: 12%		
						Butyric acid: 22%		
Olive oil mill effluent	Lipid	Digested sludge from biogas plant	7.1	0.15	0.25 gCOD_CA_/g SCOD	Acetic acid: 53%	Acetic	[29]
						Propionic acid: 15%		
						Butyric acid: 28%		
						Valeric acid: 4%		

AD—anaerobic digestion; UASB—up-flow anaerobic sludge blanket; SCOD—soluble chemical oxygen demand; VS—volatile solids; ng—not given; WWTP—wastewater treatment plant; BSG—brewer’s spent grain.

## Data Availability

The original contributions presented in this study are included in the article. Further inquiries can be directed to the corresponding author.
